# Supplementary material for: Computational Identification of Potential Multitarget Inhibitors of Nipah Virus by Molecular Docking and Molecular Dynamics
Source: Microorganisms. 2022 Jun 9;10(6):1181. doi: 10.3390/microorganisms10061181 (PMC9227315; doi:10.3390/microorganisms10061181)
Supplement: Supplementary file 1 [file microorganisms-10-01181-s001.zip › microorganisms-1673572-supplementary/Supplementary-figure S1 .pdf]

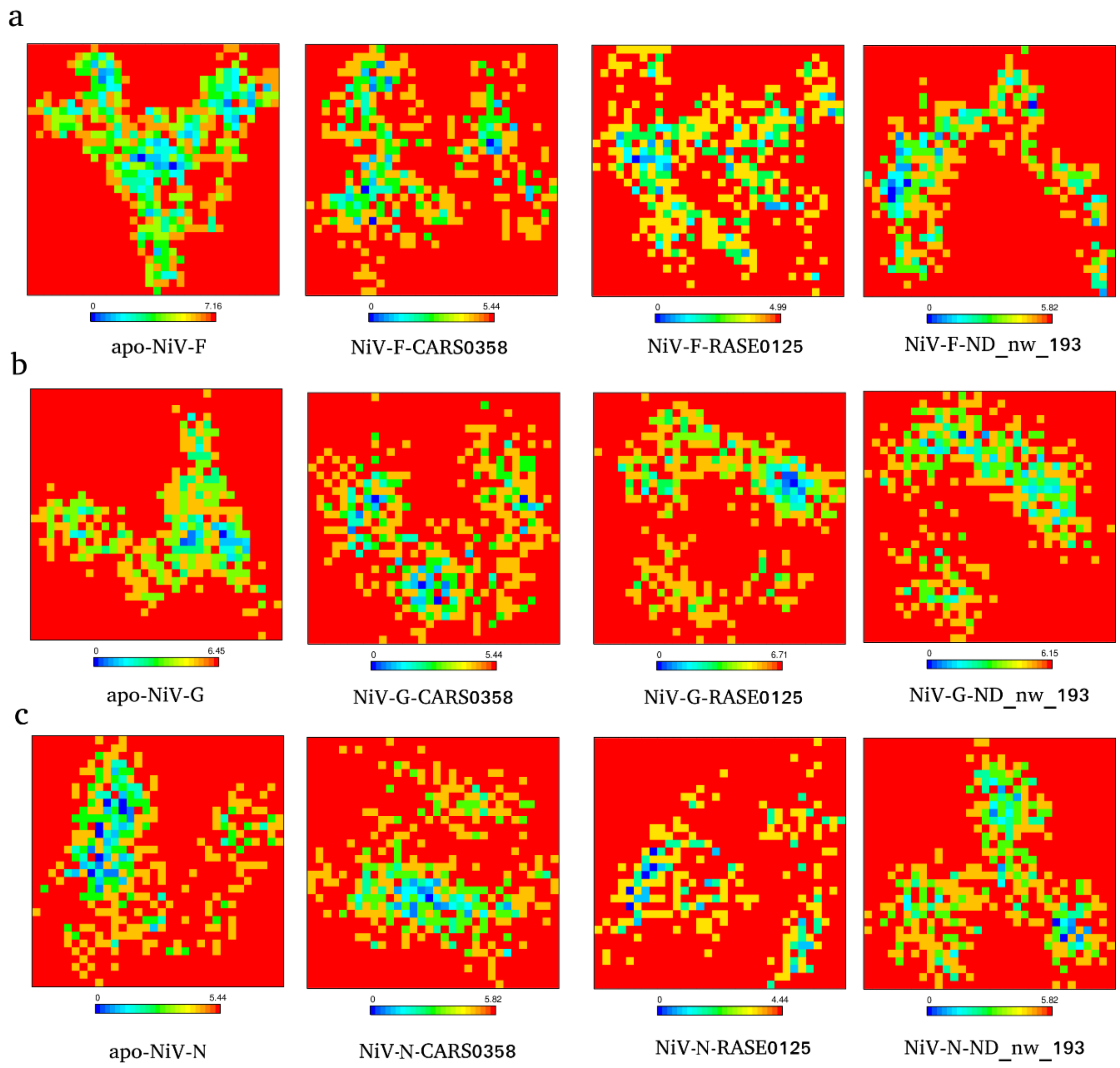

Figure S1. 2D graphs of the Gibbs free energy landscape for NiV-F (a), NiV-G (b) and NiV-N (c) (apo- and docked complexes)
